# Systolic left ventricular function is preserved during therapeutic hypothermia, also during increases in heart rate with impaired diastolic filling

**DOI:** 10.1186/s40635-015-0041-6

**Published:** 2015-02-24

**Authors:** Viesturs Kerans, Andreas Espinoza, Helge Skulstad, Per Steinar Halvorsen, Thor Edvardsen, Jan Frederik Bugge

**Affiliations:** Department of Anesthesiology, Rikshospitalet, Division of Emergencies and Critical Care, Oslo University Hospital, Postbox 4950, Nydalen, N-0424 Oslo, Norway; Institute of Clinical Medicine, Faculty of Medicine, University of Oslo, Oslo, Norway; The Intervention Centre, Rikshospitalet, Oslo University Hospital, Oslo, Norway; Department of Cardiology, Rikshospitalet, Oslo University Hospital, Oslo, Norway; Department of Research and Development, Division of Emergencies and Critical Care, Oslo University Hospital, Oslo, Norway

**Keywords:** Therapeutic hypothermia, Atrial pacing, Cardiac function, Diastolic dysfunction, Echocardiography

## Abstract

**Background:**

Systolic left ventricular function during therapeutic hypothermia is found both to improve and to decline. We hypothesized that this discrepancy would depend on the heart rate and the variables used to assess systolic function.

**Methods:**

In 16 pigs, cardiac performance was assessed by measurements of invasive pressures and thermodilution cardiac output and with 2D strain echocardiography. Left ventricle (LV) volumes, ejection fraction (EF), transmitral flow, and circumferential and longitudinal systolic strain were measured. Miniaturized ultrasonic transducers were attached to the epicardium of the LV to obtain M-mode images, systolic thickening, and diastolic thinning velocities and to determine LV pressure-wall dimension relationships. Preload recruitable stroke work (PRSW) was calculated. Measurements were performed at 38 and 33°C at spontaneous and paced heart rates, successively increased in steps of 20 up to the toleration limit. Effects of temperature and heart rate were compared in a mixed model analysis.

**Results:**

Hypothermia reduced heart rate from 87 ± 10 (SD) to 76 ± 11 beats/min without any changes in LV stroke volume, end-diastolic volume, EF, strain values, or PRSW. Systolic wall thickening velocity (S′) and early diastolic wall thinning velocity decreased by approximately 30%, making systolic duration longer through a prolonged and slow contraction and changing the diastolic filling pattern from predominantly early towards late. Pacing reduced diastolic duration much more during hypo- than during normothermia, and combined with slow myocardial relaxation, incomplete relaxation occurred with all pacing rates. Pacing did not affect S′ or PRSW at physiological heart rates, but stroke volume, end-diastolic volume, and strain were reduced as a consequence of reduced diastolic filling and much more accentuated during hypothermia. At the ultimate tolerable heart rate during hypothermia, S′ decreased, probably as a consequence of myocardial hypoperfusion due to sustained ventricular contraction throughout a very short diastole.

**Conclusions:**

Systolic function was maintained at physiological heart rates during therapeutic hypothermia. Reduced tolerance to increases in heart rate was caused by lack of ventricular filling due to diastolic dysfunction and shorter diastolic duration.

## Background

Mild (therapeutic) hypothermia (32 to 34°C) for a limited time interval (12–72 h) seems to improve neurological outcome in several clinical conditions, including patients resuscitated after out-of-hospital cardiac arrest (OHCA) [1,2]. In a recently published multi center trial on OHCA patients, however, there was no difference in neurological outcome between target temperatures of 36°C versus 33°C [3]. Compared to moderate (28 to 32°C) or deep (<28°C) hypothermia, therapeutic hypothermia is well tolerated with few side effects, and hemodynamic stability seems to be well preserved. However, therapeutic hypothermia affects cardiac performance, and the dominating effects on the normal heart may differ substantially from the effects on hearts exposed to some kind of injury. In patients with cardiogenic shock, therapeutic hypothermia has been shown to improve cardiac performance assessed by increases in thermodilution cardiac index (CI) and mean arterial pressure (MAP) [4], reduce the need for vasopressors, and improve cardiac performance assessed by echocardiography [5]. Similarly, experimental studies have shown that therapeutic hypothermia improves cardiac performance during acute myocardial infarction [6] and post resuscitation [7] and protects the heart from ischemic injury by reducing infarct size [8]. In unselected OHCA patients, however, therapeutic hypothermia reduced MAP and thermodilution CI and increased the need for vasopressors [9]. In patients with presumably normal hearts, both systolic and diastolic dysfunction with reduced cardiac output were demonstrated by echocardiography during therapeutic hypothermia [10].

Hypothermia is an integral part of cardiac surgery, and postoperative patients frequently are mildly hypothermic despite rewarming on cardiopulmonary bypass (CPB). Clinical [11] and experimental [12] studies in relation to CPB have demonstrated reduced cardiac performance during mild hypothermia which was particularly pronounced at increased heart rates. However, there are also opposite findings demonstrating improved left ventricular function after CPB in experimental animals [13].

Conflicting results also exist regarding the physiological effect of therapeutic hypothermia on the normal heart and circulation [14,15]. In isolated myocardium, isometric force development increases during hypothermia, but the optimal frequency for force development is lower compared to normothermia [16]. In the in situ mammalian heart, there is general agreement that therapeutic hypothermia impairs diastolic heart function, but the effects on systolic function and blood pressure are contradictory. The reason for this is not clear, but may be related to the experimental and clinical conditions and to the methods by which cardiac performance is assessed.

Among clinicians, however, there seem to be a widespread opinion that therapeutic hypothermia has a positive inotropic effect and improves cardiac systolic performance as long as heart rate is not increased [17,18]. In the present study, we tested this hypothesis in an open-chest porcine model. The effects of therapeutic hypothermia on the heart and circulation were measured both by traditional thermodilution and pressure-dimension relationships and by imaging techniques with 2D strain echocardiography and with small ultrasonic transducers attached to the epicardium of the left ventricle (LV) [19].

## Methods

### Preparation

A total of 16 Norwegian land race swine with mean weight of 52.2 ± 6.3 kg were included in the study. The animals were sedated by intramuscular injection of ketamine (20 mg/kg), atropine (0.02 mg/kg), and azaperone (3 mg/kg). Anesthesia was induced by intravenous (iv) injection of pentobarbital 3 mg/kg and morphine 1.0 mg/kg and maintained by iv morphine-infusion (1 to 2 mg/kg/h) and isoflurane-inhalation (1.0 to 1.5%), keeping the end-tidal concentration at the same level during normo and hypothermia within individual animals. Muscle relaxants were not used. Plasma catecholamines were measured in two animals and showed no difference between normo and hypothermia. The animals underwent a tracheotomy and were ventilated by air/oxygen (FiO_2_ = 0.4) with a tidal volume of 10 to 15 ml/kg, adjusted during hypothermia to maintain a stable PCO_2_.

An 8-French vascular introducer was placed in the ascending aorta through the carotid artery for measurements of hydrostatic blood pressure and for placement of a micromanometer (MPC-500, Millar Instruments, Houston, TX, USA) into the LV cavity for measurements of LV pressure. For cooling and rewarming of the animals, a water-circulated catheter (Cool Line®, Zoll, Chelmsford, MA, USA) was placed in the inferior vena cava from the left femoral vein and connected to a designated thermal regulation system (Coolgard 3000®, Zoll). A pulmonary artery catheter (CCO, Edwards Lifesciences, Irvine, CA, USA) was put in place from the left jugular vein to measure cardiac output (CO) and central temperature. After sternotomy, epicardial ultrasonic transducers (Imasonic SA, Besançon, France) were sutured to the anterior LV wall for continuous monitoring of wall thickness through the cardiac cycle. Two pacemaker leads were attached to the right atrial appendage. A snare was placed around the inferior caval vein for acute volume unloading. The preparation lasted for approximately 190 min followed by a minimum of 30 min of stabilization before measurements. All animals were handled according to international regulations. The study was approved by the Oslo University Hospital Institutional Animal Care and Use Commitee and was carried out in accordance with the Norwegian National Legislation on animal experimentation and the European Union Directive 2010/63/EU on the protection of animals used for scientific purposes.

### Animal model

Measurements were made at normothermia (38°C) and during mild hypothermia (33°C) at spontaneous and paced heart rates. The water-circulated catheter was used to keep stable temperatures both during normo and hypothermia. Atrial pacing was performed at 100 beats/min, and then successively increased in steps of 20 beats/min up to the toleration limit. Measurements were performed after 5 min of stable hemodynamics. At each intervention, transient constriction of the inferior caval vein was performed for acute volume unloading. The first 11 animals were tested for maximal toleration to increases in heart rate. The limit of toleration was set to the maximal frequency at which the animal had stable hemodynamics for 5 min and through the following recording period. Pacing was stopped immediately if hemodynamic deterioration occurred. In the other five animals, pacing was stopped at 140 beats/min during normothermia and 120 beats/min during hypothermia because reliable recordings during caval vein constriction were not obtainable at higher heart rates. Except for the testing of heart rate toleration limits, results are therefore presented and compared within and between temperature levels up to frequencies of 140 and 120 beats/min for normo and hypothermia, respectively. In four animals, cooling was instituted during instrumentation, and hypothermic recordings were made first, followed by rewarming and normothermic recordings. In the other animals, normothermic recordings were performed first.

### Measurements and calculations

#### Hemodynamics

**Figure 1:**
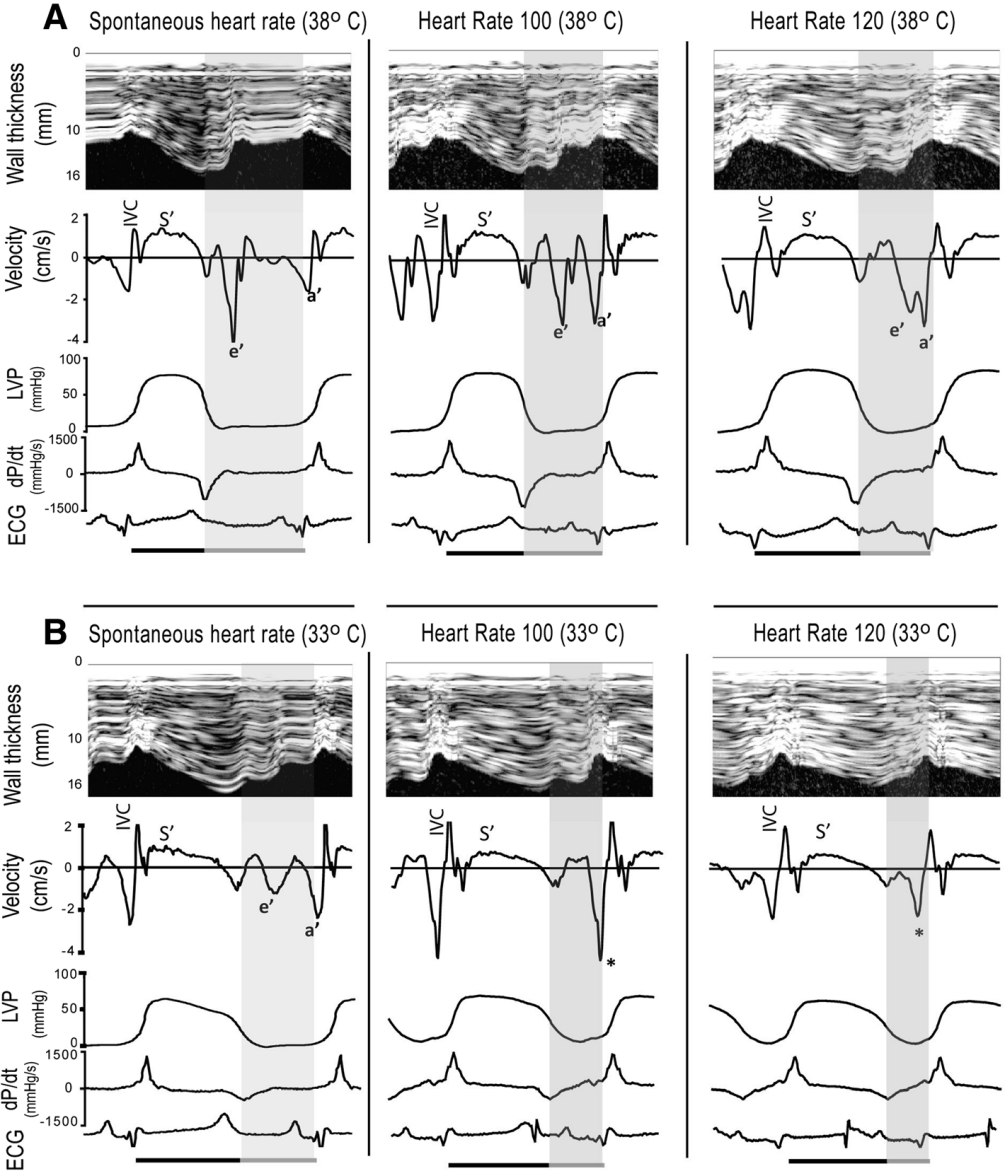
**Recordings from spontaneous and atrially paced heart rates during normo (A) and hypothermia (B)**.The upper images in each panel show M-mode recordings with corresponding wall thickening velocities synchronized with pressure measurements (LVP), dp/dt, and electrocardiogram (ECG) below. The shaded area represents diastole which is shortened both by pacing and hypothermia. Hypothermia reduced peak systolic thickening velocity (S′) and shifted the diastolic velocities from predominantly early (e′) towards late (atrial-induced) (a′). Pacing during hypothermia caused fusion of the e′ and a′ waves (marked by an asterisk). IVC, isovolumetric contraction.

CO was measured in triplets by thermodilution of 10 ml of ice-cold saline. Stroke volume (SV) was calculated as CO divided by heart rate. Peak systolic LVP, end-systolic LVP (ESP), and end-diastolic LVP (EDP) were recorded. LV afterload was estimated by arterial elastance (Ea) = ESP/SV. Peak positive and negative amplitude of the time derivative of LVP were recorded (dP/dt_max_, dP/dt_min_). Duration of systole was calculated from the onset of steep upstroke of dP/dt to dP/dt_min_. Diastolic duration was calculated from dP/dt_min_ to onset of systole (Figure 1). The LV relaxation time constant (τ) was calculated during isovolumic relaxation time according to Weiss et al. [20]. Time for complete relaxation was defined as 3.5τ from end systole [21] and was calculated as a fraction of diastolic duration (3.5τ/DD).

### Echocardiography

A Vivid 7 scanner with a 2.5/2.75 MHz probe (GE Vingmed Ultrasound, Horten, Norway) was used directly on the heart with a gel stand off for 2D echocardiography/Doppler recordings. End-diastolic volumes (EDV) were calculated by Simpson biplane method. Ejection fraction (EF) was calculated as SV/EDV. Longitudinal and circumferential strain in septum and lateral wall mid-segments were calculated by speckle tracking in 2D grey scale images (frame rate 63 ± 11). Transmitral flow was measured from Doppler recordings with assessments of early (E) and late (A) (atrial-induced) diastolic filling velocities.

### Ultrasonography

Wall thickness was measured through the cardiac cycle by M-mode recordings from the epicardial ultrasonic transducers (Figure 1). This allowed precise measurements from the exact same region during all interventions. End-systolic and end-diastolic wall dimensions were recorded. Peak systolic wall thickening velocity during ejection (S′) and early and late (atrial-induced) diastolic wall thinning velocities, (e′) and (a′) respectively, were measured as previously described [22].

Wall thickness through the cardiac cycle was plotted towards LVP resulting in pressure-wall thickness (P-WTh) loops for calculation of regional work. Regional preload recruitable stroke work (PRSW) was calculated as the relationship between P-Wth loop area and end-diastolic WTh (EDWTh) during caval constrictions.

### Statistical analysis

Data are reported as mean ± SD. The data from 38 and 33°C at spontaneous and paced heart rates were compared using paired *t*-test. To study the effect from the interventions, the data were analyzed using a linear mixed model to handle the dependencies introduced by repeated measurements within in each subject. The data were analyzed using temperature level and heart rate (HR) (spontaneous or atrially paced) as determinants in a model where variable = *β* + *β*_*1*_ (temp) + *β*_*2*_ (HR) + *β*_*3*_ (temp × HR). Time of recording was included as a covariate to account for the order of measurements and assess the effect of time. Model selection was made choosing the covariance structure with the lowest Akaike information criteria. Predicted values and residuals were inspected for goodness of model fit. Differences were considered significant if *p* ≤ 0.05.

## Results

Recordings of good quality were obtained from all experiments except from caval constriction recordings in four animals. The cooling period lasted about 100 min, while warming in the 'cold first' animals lasted 220 min on average. The average duration of each experiment was 560 min. The effects of temperature and heart rate changes were not dependent on the order of the temperature level.

### Spontaneous heart rate

The results are summarized in Tables 1 and 2 and Figure 1. Reducing the temperature from 38 to 33°C decreased the spontaneous heart rate from 87 ± 10 to 76 ± 11 beats/min. SV, EF, longitudinal and circumferential strain, PRSW, and EDV did not change. There was a small increase in EDWth. S′ and dP/dt_max_ decreased, demonstrating slower myocardial contraction. Systolic duration increased, and there was a small increase in end-systolic wall thickness (ESWth). ESP and Ea decreased, reflecting a reduction in left ventricular afterload during hypothermia, which also was reflected by a reduction in P-Wth loop area. The peak LVP, MAP, and CO decreased while EDP increased, and systemic vascular resistance (SVR) did not change.

**Table 1:** Hemodynamic and systolic variables

	**Spontaneous heart rate**			**Paced heart rate (100)**			**Paced heart rate (120)**			**Paced heart rate (140)**
**Variable**	**38°C**	**33°C**	***p***	**38°C**	**33°C**	***p***	**38°C**	**33°C**	***p***	**38°C**
Heart rate (bpm)	87 ± 10	76 ± 11	<0.001	100 ± 0	100 ± 0		120 ± 0	120 ± 0		140 ± 0
CO (l/min)	4.8 ± 0.8	3.7 ± 0.7	<0.001	5.3 ± 0.6	4.0 ± 0.8*	<0.001	5.6 ± 0.7**	3.5 ± 1.1	<0.001	5.5 ± 0.7**
SV (ml)	54 ± 7	49 ± 10	0.09	53 ± 6	40 ± 8**	<0.001	47 ± 6**	29 ± 10**	<0.001	39 ± 5**
EF (%)	69 ± 9	66 ± 11	0.40	70 ± 10	62 ± 10*	0.12	67 ± 8	50 ± 16**	<0.001	61 ± 5**
MAP (mmHg)	62 ± 9	54 ± 9	0.016	65 ± 9	54 ± 7	0.008	66 ± 8	52 ± 9	<0.001	66 ± 10
SVR (dyn × s/cm^5^)	900 ± 165	978 ± 179	0.26	866 ± 118*	934 ± 144	0.58	840 ± 143*	1047 ± 387	<0.01	843 ± 172*
pLVP (mmHg)	85 ± 7	68 ± 10	<0.001	85 ± 7	67 ± 10	<0.001	84 ± 6	64 ± 11**	<0.001	83 ± 7
dp/dt_max_ (mmHg/s)	1372 ± 494	1111± 381	0.02	1303 ± 199	1153 ± 316	0.01	1337 ± 321	1203 ± 361	0.05	1294 ± 293
ESP (mmHg)	43 ± 7	33 ± 8	0.005	44 ± 6	30 ± 8*	<0.001	41 ± 7	29 ± 7*	<0.001	40 ± 10*
Ea (mmHg/ml)	0.8 ± 0.2	0.6 ± 0.2	0.002	0.8 ± 0.2	0.7 ± 0.3	0.009	0.9 ± 0.3	0.9 ± 0.3*	0.84	1.2 ± 0.4**
Strain_l_ (%)	−19.7 ± 2.6	−18.3 ± 3.8	0.21	−19.1 ± 3.8	−14.8 ± 2.4**	<0.001	−16.4 ± 2.6**	−12.7 ± 3.4**	<0.001	−13.2 ± 2.6**
Strain_c_ (%)	−18.6 ± 3.8	−17.2 ± 3.4	0.21	−17.6 ± 3.0	−14.3 ± 3.3**	0.007	−15.2 ± 2.4**	−12.1 ± 3.1**	0.004	−12.7 ± 2.1**
S′ (cm/s)	1.1 ± 0.3	0.7 ± 0.2	<0.001	1.1 ± 0.3	0.7 ± 0.2	<0.001	1.1 ± 0.3	0.7 ± 0.3	<0.001	1.0 ± 0.4
SysD (ms)	338 ± 17	465 ± 32	<0.001	322 ± 12**	397 ± 32**	<0.001	301 ± 19**	368 ± 28**	<0.001	274 ± 22**
ESWth (mm)	14.5 ± 1.9	15.2 ± 1.8	0.01	14.7 ± 1.8	15.2 ± 2.0	0.1	14.9 ± 1.8	15.8 ± 2.2	0.006	14.3 ± 1.9
P-Wth loop (mm × mmHg)	14 ± 5	11 ± 5	0.016	12 ± 5*	8 ± 4**	0.001	11 ± 4**	6 ± 3**	<0.001	9 ± 3**

Values are expressed as mean ± SD. *p ≤ 0.05 for comparison with spontaneous heart rate within temperature level. **p ≤ 0.01 for comparison with spontaneous heart rate within temperature level. CO, cardiac output; SV, stroke volume; EF, ejection fraction; MAP, mean arterial pressure; SVR, systemic vascular resistance; pLVP, peak left ventricular pressure; ESP, end-systolic pressure Ea, arterial elastance; Strain_l_, longitudinal strain; Strain_c_, circumferential strain; S′, peak systolic wall thickening velocity; SysD, systolic duration; ESWth, end-systolic wall thickness; P-Wth loop, left ventricular pressure-wall thickness loop area.

**Table 2:** Diastolic variables

	**Spontaneous heart rate**			**Paced heart rate (100)**			**Paced heart rate (120)**			**Paced heart rate (140)**
**Variable**	**38°C**	**33°C**	***p***	**38°C**	**33°C**	***p***	**38°C**	**33°C**	***p***	**38°C**
EDP (mmHg)	10 ± 3	12 ± 2	0.004	10 ± 3	12 ± 3	0.09	10 ± 2	13 ± 4	0.002	12 ± 3
EDV (ml)	79 ± 10	75 ± 11	0.26	77 ± 11	64 ± 9**	0.002	69 ± 8**	57 ± 6**	<0.001	64 ± 8**
EDWth (mm)	10.6 ±1.5	11.1 ± 1.5	0.003	11.0 ± 1.4**	11.9 ± 2.0*	0.02	11.1 ± 1.5**	12.4 ± 2.2**	0.02	11.0 ± 2.0**
Tau (s)	31 ± 6	54 ± 12	<0.001	35 ± 8	61 ± 18	0.001	29 ± 4	53 ± 13	<0.001	29 ± 4
dp/dt_min_ (mmHg/s)	−1385 ± 529	−742 ± 328	<0.001	−1190 ± 437	−773 ± 257	0.005	−1324 ± 426	−670 ± 332**	<0.001	−1476 ± 555
e' (cm/s)	−2.5 ± 0.9	−1.6 ± 0.7	0.002	−2.3 ± 0.5			−1.9 ± 0.6			
DD (ms)	319 ± 79	299 ± 88	0.26	281 ± 11*	199 ± 35**	<0.001	199 ± 14**	142 ± 25**	<0.001	148 ± 23**
DD/CycleD	0.48 ± 0.05	0.38 ± 0.07	<0.001	0.47 ± 0.02	0.33 ± 0.04**	<0.001	0.40 ± 0.03**	0.28 ± 0.05**	<0.001	0.34 ± 0.05**
3.5tau/DD	0.36 ± 0.11	0.72 ± 0.35	0.001	0.44 ± 0.10**	1.10 ± 0.32**	<0.001	0.52 ± 0.09**	1.38 ± 0.49**	<0.001	0.69 ± 0.15**

Values are expressed as mean ± SD. *p ≤ 0.05 for comparison with spontaneous heart rate within temperature level. **p ≤ 0.01 for comparison with spontaneous heart rate within temperature level. EDP, end-diastolic pressure; EDV, end-diastolic volume; EDWth, end-diastolic wall thickness; e′, early wall thinning velocity; DD, diastolic duration; DD/CycleD, diastolic fraction of cardiac cycle duration.

**Figure 2:**
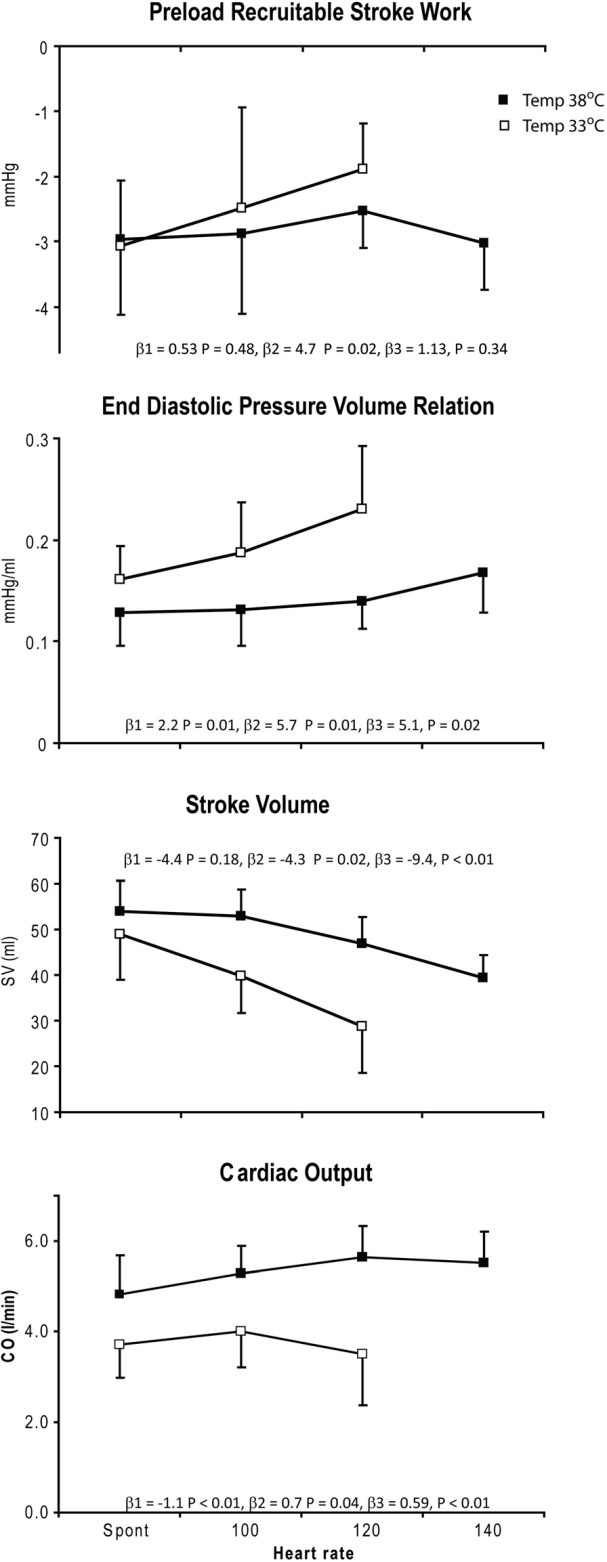
**PRSW, EDPVR, SV, and CO.** Changes in preload recruitable stroke work, end-diastolic pressure volume relation, stroke volume, and cardiac output at spontaneous and paced heart rates (HR) during normo (■) and hypothermia (□). The effect of HR and temperature were analyzed in a mixed model analysis in which variable = β + β_1_ (temperature) + β_2_ (HR) + β_3_ (temperature × HR). Coefficients and *p* values are given for each variable.

The diastolic changes have been reported in detail previously [22]. In summary, the absolute value of dP/dt_min_ and e′ decreased, whereas τ increased, reflecting slow and prolonged myocardial relaxation. The relative reduction in e′ was not significantly different from the relative reduction in S′, 35 ± 21 and 28 ± 15% (*p* = 0.24), respectively. There was a shift from predominately early to predominantly late ventricular filling with reversion of the E/A relationship (data not shown), and there was an increase in left ventricular stiffness [22], reflected by an increase in end-diastolic pressure-volume relationship (EDPVR) (Figure 2). Diastolic duration remained unchanged, making the diastole occupy a shorter part of the cardiac cycle, but allowed enough time for complete relaxation (3.5τ/DD < 1).

### Paced heart rates

During normothermia, SV successively decreased at heart rates above 100 beats/min, and this was reflected by increases in EDWth at constant ESWth (Tables 1 and 2). The systolic variables PRSW, dP/dt_max_, and S′ did not change with increasing heart rates, whereas EF decreased at higher heart rates. During normothermia, CO increased slightly with increasing heart rates up to 120 beats/min and started to decline at heart rates above 140 beats/min. Peak LVP and MAP did not change, whereas SVR slightly decreased with increasing heart rates up to 140 beats/min (Table 1). ESP and Ea, however, were unchanged up to a frequency of 120 beats/min. ESP decreased, and Ea increased when heart rate was increased to 140 beats/min. EDP did not significantly change whereas EDV successively decreased at heart rates above 100 beats/min, making significant increases in EDPVR at higher heart rates (Figure 2).

During hypothermia, SV decreased successively with increases in heart rate, and in the mixed model analysis, heart rate had a significantly greater negative effect on SV during hypo than during normothermia (Figure 2). The changes in SV were reflected by increases in EDWth without changes in ESWth (Tables 1 and 2). PRSW did not change significantly in the mixed model analysis (Figure 2), but trended towards a decrease. S′ and dP/dt_max_ did not change upon increases in heart rate up to 120 beats/min. EF decreased successively (Table 1). CO slightly increased when heart rate was increased to 100 beats/min, but decreased with further increases in heart rate (Table 1). MAP was not significantly changed whereas peak LVP slightly decreased when heart rate was increased to 120 beats/min. SVR did not change, whereas Ea slightly increased with increasing heart rates. EDP did not significantly increase, but EDV decreased successively with a significantly greater effect on the EDPVR during hypo than normothermia (Figure 2).

Both systolic and diastolic duration, diastolic fraction of the cardiac cycle, and the absolute values of longitudinal and circumferential strain decreased with increasing heart rate, but significantly more during hypothermia (Tables 1 and 2). The decreases in strain paralleled the decreases in diastolic filling (EDV and EDWth) and SV. The absolute value of dP/dt_min_ did not change during pacing at normothermia, but decreased when heart rate was increased to 120 beats/min during hypothermia. τ, however, was not influenced by heart rate neither during normo nor hypothermia. Due to fusion of the e′ and a′ waves (Figure 1), e′ could not be measured during pacing at hypothermia. During normothermia, the e′ and a′ waves were still separated at a frequency of 100 and partly separated at 120 beats/min, and e′ was not significantly changed by pacing (Table 2). During normothermia, 3.5τ/DD < 1 at all comparable pacing rates allowing enough time to complete relaxation. During hypothermia, however, 3.5τ/DD exceeded unity at all pacing rates, making the diastole to short for complete relaxation and filling (Table 2).

**Figure 3:**
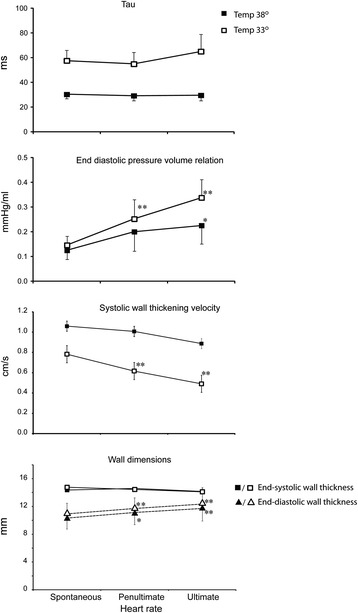
**Tau, end-diastolic pressure volume relation, systolic wall thickening velocity, and left ventricular wall dimensions.** Changes in tau, end-diastolic pressure volume relation, systolic wall thickening velocity, and left ventricular wall dimensions during spontaneous, penultimate, and ultimate tolerable heart rates at normo- (closed symbols) and hypothermia (open symbols). *p ≤ 0.05 for comparison with spontaneous heart rate within temperature level. ** *p* ≤ 0.01 for comparison with spontaneous heart rate within temperature level.

The tolerance to increases in heart rate was significantly reduced during hypothermia (*p* = 0.01). At normothermia, all 11 pigs tolerated a paced frequency of 140, seven pigs tolerated 160, and four tolerated 180 beats/min. During hypothermia, all pigs tolerated 120 beats/min, eight pigs tolerated 140, and three tolerated 160 beats/min. Because of variations in heart rate tolerability between individual animals, we made comparisons with penultimate and ultimate tolerable heart rates to detect physiological changes occurring close to the toleration limit both during normo and hypothermia. These data are shown in Figure 3. S′ significantly decreased both at penultimate and ultimate heart rates during hypothermia, but there were no statistically significant decrease during normothermia (*p* = 0.20). ESWTh was unchanged during normothermia (*p* = 0.54), and the decrease during hypothermia was not statistically significant (*p* = 0.11). There was a further, but not statistically significant (*p* = 0.15), increase in τ at the ultimate heart rate during hypothermia, reflecting further reduction in relaxation velocity parallel to the reduction in contraction velocity. Taken together with a further increase in EDPVR, this indicates maintained ventricular contraction throughout diastole.

## Discussion

Systolic contraction velocity was slowed during therapeutic hypothermia, but compensated for by prolongation of systolic duration, and systolic function assessed by ESWth (end-systolic volume (ESV)) and PRSW was well preserved, but not increased at any heart rate. Therapeutic hypothermia impaired the tolerance to increasing heart rates induced by atrial pacing, by impeding diastolic filling due to slow relaxation and successive shortening of diastolic duration. Pacing itself had no effect on myocardial velocities, except during hypothermia at heart rates close to the toleration limit, where contraction and relaxation velocities further decreased. The latter is probably related to impaired myocardial perfusion due to shortness of diastolic duration and incomplete diastolic relaxation with persisting myocardial contraction at end diastole.

### Spontaneous heart rate

The kinetics of cardiac contraction and relaxation are tightly linked during different physiological conditions [23], and the parallel decreases in systolic and diastolic velocities during hypothermia are in agreement with these findings.

During hypothermia, the slower myocardial contraction measured by systolic wall thickening velocity was compensated for by prolonged systolic duration, allowing enough time to maintain SV, EF, and strain. Reduced afterload may have facilitated ventricular ejection as indicated by a small increase in ESWth and thus masked a systolic dysfunction, but no change in the load independent variable PRSW indicated preserved pump function. This is in contrast to improved systolic function found by Post and co-workers [14]. Going into their study in more detail reveals that their conclusion of increased inotropy at 33°C was based on a leftward shift of the end-systolic pressure volume relationship (end-systolic elastance) during ventricular unloading at a paced heart rate of 100 beats/min. There was no change in PRSW. Similar to our present and previous study [22], they found that hypothermia increased passive ventricular stiffness and increased ventricular stiffness increases end-systolic elastance even at depressed contractility [24]. Increased ventricular stiffness may also resist dynamic ventricular contraction and thus obscure a hypothermia-induced increase in contractile force.

This may, at least partly, explain the difference between the in situ heart and isolated cardiac trabeculae which have little supportive tissue and where only isometric contractile force is measured [16].

Although slow relaxation was not compensated for by a corresponding increase in diastolic duration, there was enough time for complete relaxation and filling demonstrated by 3.5τ/DD < 1 and maintained EDV. EDWth, however, increased slightly, indicating a small reduction in diastolic filling that could not be detected by the biplane Simpson method. Increased wall stiffness is probably the cause of this small decrease in diastolic filling and is supported by the increase in EDPVR.

### Paced heart rates

PRSW was not significantly reduced during pacing, and the tolerance to increases in heart rate seemed mainly to be limited by diastolic filling. Atrial pacing not only reduced diastolic duration but also successively reduced the diastolic fraction of the cardiac cycle, and both absolute and relative diastolic shortening were accentuated by hypothermia (Table 2). Time for complete relaxation (3.5τ/DD < 1) was achieved during pacing at normothermia, but incomplete filling with reduced EDV and SV occurred at higher heart rates. During hypothermia, incomplete relaxation (3.5τ/DD > 1) and filling occurred at all pacing rates with a much greater impact on EDV and SV than during normothermia (Figure 2 and Table 2). The changes in longitudinal and circumferential strain paralleled the changes in EDV and SV in agreement with the findings of others [25]. These changes were accompanied by reductions in EF which occurred already at a heart rate of 100 beats/min during hypothermia. During normothermia, a significant reduction in EF was observed at 140 beats/min. Clinically, EF is mainly used to evaluate systolic performance, and a reduced EF is a measure of systolic dysfunction. In the present setting, however, it mainly represents diastolic filling. The ESWTh was unchanged during pacing at both temperatures indicating adequate emptying of the LV and a constant ESV. At constant ESV, EF is only dependent on EDV and implies that any reduction in EDV will result in a reduction in EF. Comparing the frequency of 100 beats/min during hypothermia with the frequency of 140 during normothermia demonstrates similar EDV, SV, and hence EF.

**Figure 4:**
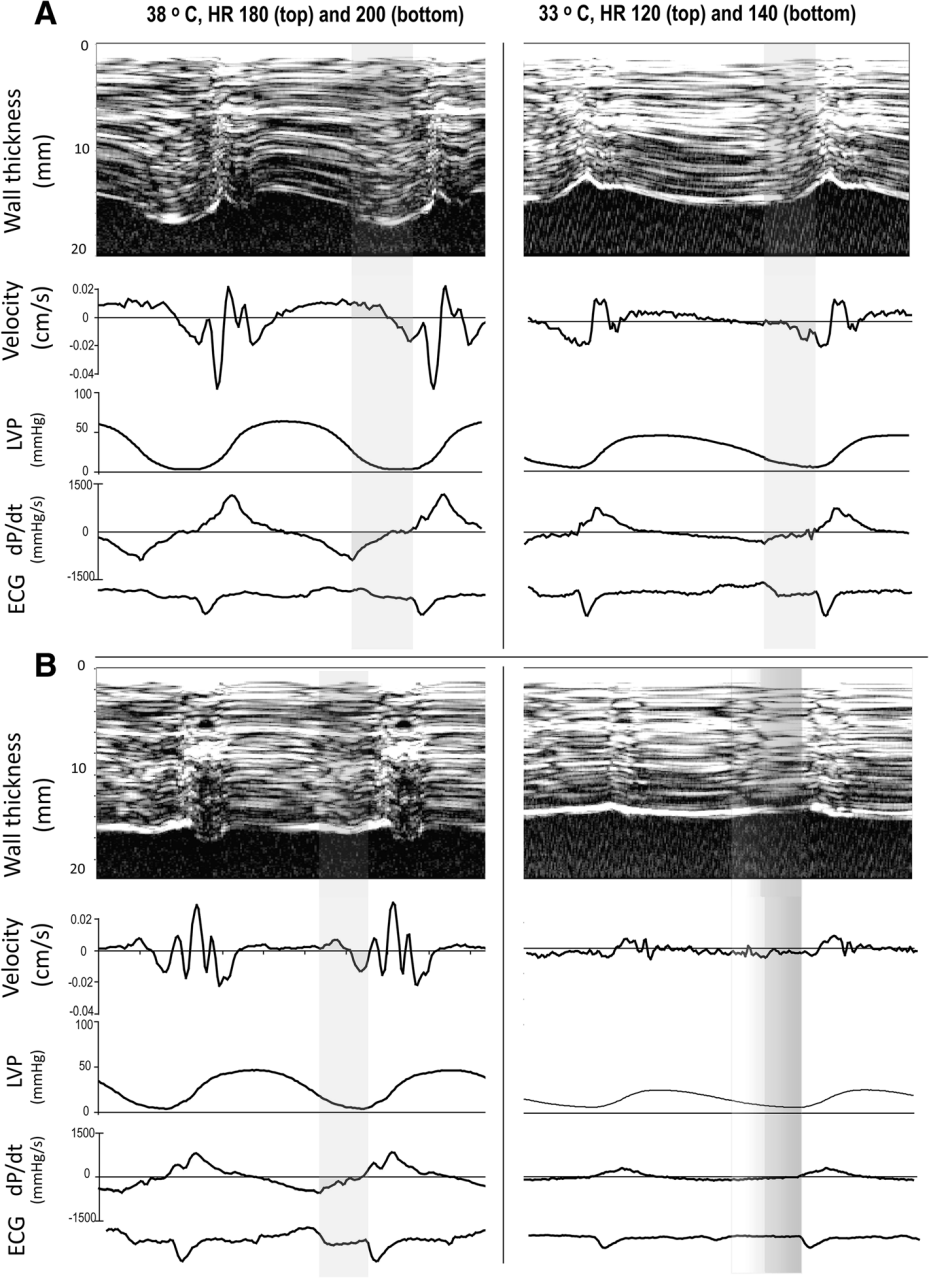
**Recordings from the ultimate tolerable (A) and the following not tolerable heart rate (B) during normo (left) and hypothermia (right).** For explanation, see Figure 1.

With the possible exception for the ultimate tolerable heart rate, the slower S′ during hypothermia did not significantly affect systolic emptying, demonstrating that systolic duration was long enough to adequately empty the heart from the actual EDV. This prolongation of systole at the expense of diastole during hypothermia seems to be an inappropriate response to pacing induced increases in heart rate. Successive shortening of diastolic duration with incomplete relaxation and persisting myocardial contraction throughout diastole as demonstrated by successive increases in EDPVR (Figure 3) could jeopardize myocardial perfusion and thus be responsible for the further slowing of S′ at the penultimate and ultimate tolerable heart rates and the parallel increase in τ. This is in accordance with results from isolated myocardial muscle strips from both experimental animals and explanted failing human hearts where increased diastolic force was demonstrated during hypothermia [16,26]. In explanted human hearts at a temperature of 33°C, increased diastolic force occurred already at a frequency of 1 Hz [26]. In experimental animals, diastolic force occurred at supra-physiological frequencies accompanied by reductions in developed force during systole [16] which approximated zero as diastolic force continued to increase towards ultimate tolerable heart rates [27]. EDP did not change in the present study, and the increase in EDPVR is only due to reduced EDV. Diastolic LVP has to be lower than peak atrial pressure for the mitral valve to open, and diastolic filling approaches zero when these two pressures almost equals during ultra-short diastolic duration and maintained diastolic contraction. An M-mode picture of the not tolerated heart rate is shown in Figure 4 demonstrating almost absence of diastolic wall thinning both during normo and hypothermia.

### Methodological considerations

The regional measurements from the epicardial transducers were used to estimate global myocardial changes. There is no data to indicate that therapeutic hypothermia should have different effects in various regions of the healthy myocardium and there was accordance between the epicardial and global 2D echocardiographic recordings as also demonstrated previously [22]. The parallel changes in strain and SV also support the global nature of the hypothermic changes. The epicardial transducer was also used to obtain serial recordings during transient volume unloading, and PRSW were estimated from these recordings. Glower and co-workers [28] demonstrated highly linear relationships between stroke work and preload whether preload was measured as global EDV or regional end-diastolic segment length by sonomicrometry. Even if the magnitude of systolic wall thickening is substantially greater than segment length shortening [29], the linearity of responses to variations in preload was equivalent (*r*^2^ = 0.88 ± 0.11), making the P-Wth loops good estimates of myocardial work.

### Clinical implications

Cardiac performance was well maintained during hypothermia at spontaneous heart rate, but at a less afterload and, hence, stroke work. Reduced workload may be important for the injured or failing heart, and therapeutic hypothermia may thus have favorable effects on both cardiac and systemic oxygen supply-demand balance and contribute to the good outcome observed in patients with cardiogenic shock after OHCA [30]. Similarly, for hearts suffering from post cardiac surgery dysfunction, therapeutic hypothermia for the first postoperative hours might be beneficial provided that heart rate is not inadequately increased by pacing. Even not intended, patients are often mildly hypothermic immediately after cardiac surgery. These patients are often paced by an epicardial pacemaker when bradycardia occurs. The present study indicates that mild bradycardia is probably acceptable. One should, however, be cautious to increase the frequency above the normal resting heart rate range.

The Doppler recordings demonstrated diastolic dysfunction at spontaneous heart rate, and 2D echocardiography showed reduced EF at increased heart rates during hypothermia. These findings indicate that interpretation of echocardiographic assessments of cardiac function is more complicated in hypothermic patients. The echocardiographic findings may at least partly be due to the hypothermia itself and will be reversed during rewarming.

### Limitations

We did not obtain pre-hypothermia and post-hypothermia recordings at 38°C in each animal, but we did perform the intervention in reverse order in four of the animals. The effects of hypothermia returned to normal after warming to 38°C, and the order of the intervention did not prove significant in the mixed model analysis. Deep hypothermia reduces both systolic and diastolic function, and in a similar pig model, systolic function was only partially reversed after rewarming from 25°C. Diastolic function, however, reversed completely [31]. In our study, systolic performance estimated by PRSW was minimally affected by hypothermia, and thus, little to reverse. Both systolic and diastolic velocities increased to anticipated values when the four animals were warmed from therapeutic hypothermia.

Deep anesthesia reduces the sympathetic response to hypothermia, and no changes in plasma catecholamines were observed, similar to the findings of Filseth and co-workers [31]. However, we do not know if the cardiodepressive effects of anesthesia are different during hypothermia. The use of volatile anesthetics is based on continuous measurements of alveolar concentrations and is easily adjusted. Hence, it is not influenced by metabolic effects of hypothermia, but the plasma morphine concentrations may have increased despite adjustments. However, deep anesthesia including opiates is routinely used during therapeutic hypothermia after OHCA and during cardiac surgery, making this experimental study close to these clinical settings.

## Conclusions

Therapeutic hypothermia during anesthesia reduces velocities of contraction and relaxation. Slow contraction is compensated for by prolongation of systole at all heart rates maintaining adequate emptying of the LV, and the ability to generate work is not reduced. Slow relaxation leads to diastolic dysfunction with reduced tolerance to pacing induced increases in heart rate because of reduced diastolic filling and sustained diastolic contraction at higher heart rates. Echocardiographic measurements of cardiac function in hypothermic patients should be interpreted cautiously because apparent systolic and diastolic dysfunction may at least partly, be due to effects caused by transient hypothermia.

For the following variables, a more detailed explanation is given as follows:

- Abbreviation/variable/measurement/meaning
- Ea/arterial elastance/calculated: Ea = ESP / SV/estimation of LV afterload
- EF/ejection fraction/calculated: EF = SV / EDV/the fraction of EDV ejected during systole
- EDV/end-diastolic volume/2D echocardiography (Simpson's biplane method)/marker of cardiac filling at end of diastole.
- EDWth/end-diastolic wall thickness/ultrasonography (miniaturized transducer)/LV wall thickness at end of diastole as a marker of cardiac filling
- ESWth/end-systolic wall thickness/ultrasonography (miniaturized transducer)/LV wall thickness at end of systole as a marker of systolic emptying of LV
- P-Wth loop/pressure-wall thickness loop/micromanometer and ultrasonography/the relationship between LV pressure and wall thickness through one cardiac cycle. The loop area is an estimate of regional myocardial stroke work
- PRSW/preload recruitable stroke work/micromanometer and ultrasonography/the relationship between EDWth and P-Wth loop area during diastolic unloading (caval vein constriction) as a marker of myocardial force (inotropy).
- e′/early LV wall thinning velocity/ultrasonography (miniaturized transducer)/LV wall thinning velocity during early diastolic filling as a marker of diastolic function
- a′/late LV wall thinning velocity/ultrasonography (miniaturized transducer)/LV wall thinning velocity during atrial contraction
- S′/peak systolic LV wall thickening velocity/ultrasonography (miniaturized transducer)/a marker of contractile function
- τ/tau: left ventricular relaxation time constant/micromanometer/a measure of LV myocardial relaxation during diastole

## Abbreviations

CI: cardiac index
CO: cardiac output
CPB: cardiopulmonary bypass
DD: diastolic duration
EDP: end-diastolic pressure
ESP: end-systolic pressure
HR: heart rate
LV: left ventricle
LVP: left ventricular pressure
MAP: mean arterial pressure
OHCA: out-of-hospital cardiac arrest
SV: stroke volume
